# The Association of Health Literacy with Intention to Vaccinate and Vaccination Status: A Systematic Review

**DOI:** 10.3390/vaccines10111832

**Published:** 2022-10-29

**Authors:** Leonardo Maria Siena, Claudia Isonne, Antonio Sciurti, Maria Roberta De Blasiis, Giuseppe Migliara, Carolina Marzuillo, Corrado De Vito, Paolo Villari, Valentina Baccolini

**Affiliations:** Department of Public Health and Infectious Diseases, Sapienza University of Rome, 00185 Rome, Italy

**Keywords:** health literacy, vaccination, systematic review, vaccine

## Abstract

Despite health literacy (HL) being recognized as a driver of health-promoting behavior, its influence on the vaccination decision-making process remains unclear. This study summarized current evidence on the association between HL and both intention to vaccinate and vaccination status. We searched PubMed, Scopus, and Web of Science, retrieving observational studies published until January 2022 that used HL-validated tools to investigate the above associations for any vaccine. Quality was assessed using the Newcastle–Ottawa scale. Twenty-one articles were included; of these, six investigated the intention to vaccinate and the remainder vaccination status. Articles on intention looked at SARS-CoV-2 vaccination using heterogeneous HL tools and were of high/fair quality. Vaccination status, mainly for influenza or pneumococcal vaccines, was explored using various HL tools; the quality was generally high. We found inconsistent results across and within vaccine types, with no clear conclusion for either vaccination intention or status. A weak but positive association was reported between a high HL level and influenza vaccination uptake for individuals aged more than 65 years. HL did not seem to significantly influence behavior towards vaccination. Differences in the methods used might explain these results. Further research is needed to investigate the role of HL in the vaccination decision-making process.

## 1. Introduction

Vaccines are one of the most effective and cost-effective tools for the primary prevention of infectious diseases [1]. They provide immunity against various illnesses, preventing death and disability in vaccinated individuals, but also protecting those who cannot be immunized by the attainment of herd immunity [2]. Despite their unquestionable success [3], immunization coverage for several diseases has plateaued or even dropped over the last decade [4]. In Europe, for example, only a few countries have reached the immunization coverage target of 95% against measles [5], and none has achieved an uptake of 90% for the final dose of the human papillomavirus (HPV) vaccine [6]. Key challenges to the attainment of such immunization goals have been unequal access to vaccine services, which particularly affects vulnerable populations, and vaccine hesitancy, a phenomenon that has attracted worldwide interest [7,8,9]. In addition, the COVID-19 pandemic has negatively impacted routine immunization services in most countries, interrupting vaccination demand and supply [10], with consequences yet to be fully quantified [11].

In this context, identifying the factors that might influence vaccination uptake has been the subject of intense research [9,12,13,14,15]. One possible factor is health literacy (HL), which is a driver of population empowerment that may contribute to a reduction in health inequality [16,17,18]. Health literacy is also inextricably linked to the social and cultural context, which includes education, and it depends on the healthcare system organization, with all these aspects varying across countries [17]. A recent update to the definition of HL has emphasized its role in improving the health and well-being of people, underlining the importance of individual engagement in reaching this goal and acknowledging the fact that organizations need to address it equitably [19,20]. Besides being considered an independent determinant of an individual’s health, HL also has a role in mediating the association between socioeconomic status and specific health outcomes, health-related behavior, and access to and use of health services [19,21]. Indeed, it has been demonstrated that people with low HL levels more frequently have poor health outcomes, such as increased hospitalization, higher rates of medication nonadherence, and a lower uptake of preventive interventions [21].

Nevertheless, while HL is a predictor of participation in cancer screening programs [22], a clear relationship between HL and vaccination behavior has not yet emerged [23]. In fact, despite the growing number of studies that have investigated the influence of HL on an individual’s behavior towards vaccination [23,24], there are few reports of HL being a determinant [23]. In addition, factors including heterogeneity in the outcomes investigated and the instruments used for assessment, as well as differences in the vaccine type and the target populations, have made it difficult to generalize the results [23,25,26]. Therefore, the aim of this systematic review was to update and summarize findings on the association between HL and vaccination behavior. We considered both intention to vaccinate and vaccination status, aiming to provide a comprehensive picture of the vaccination decision-making process and to support the implementation of public health strategies that promote vaccination uptake.

## 2. Materials and Methods

This study was performed according to the Cochrane Handbook for Systematic Reviews and the Preferred Reporting Items for Systematic Reviews and Meta-Analyses (PRISMA) statement [27,28]. The review protocol was registered at PROSPERO (identifier CRD42022302724). Because this study did not involve primary data collection, the protocol was not submitted for institutional review board approval and did not require informed consent.

### 2.1. Search Strategy and Study Selection

Three reviewers searched the bibliographic databases PubMed, Web of Science and Scopus using the following search string: (“health” AND “literacy”) OR (“health literacy” OR “health literacy [MeSH Terms]”) AND (“vaccin*” OR “immuniz*” OR “immunis*” OR “vaccination [MeSH Terms]” OR “vaccines [MeSH Terms]”). The string was adapted to fit the search criteria of each database (Supplementary Table S1). The search was conducted among reports published from database inception to 11 January 2022. No language or date restriction was applied. Duplicate articles were removed, and the title and abstract of all retrieved records were screened. Studies that did not meet the inclusion criteria were excluded. Full texts of potentially relevant articles were examined by three researchers. Disagreements were resolved through discussion and reasons for exclusion were recorded.

### 2.2. Inclusion and Exclusion Criteria

We included studies with the following characteristics: (i) reported in English or Italian, based on co-author language abilities; (ii) cross-sectional, case–control or cohort studies; (iii) investigated HL using a validated tool; (iv) provided raw data, unadjusted or adjusted estimates of the association between HL and vaccination intention and/or status in any population(s). Any statistical analysis was considered eligible. According to Sorensen at al., we adopted the following HL definition: “[people’s ability] to make judgements and take decisions in everyday life concerning healthcare, disease prevention and health promotion to maintain or improve their quality of life“ [29]. Articles that analyzed HL with non-validated tools, investigated only specific HL (e.g., oral HL), focused on specific subdomains only, or in which data or estimates of the associations of interest were not retrievable were excluded.

### 2.3. Data Collection and Quality Assessment

For each record included, three reviewers independently extracted the following information using a standardized data abstraction form: first author, year of publication, country, study design, main characteristics of the target population (age, ethnicity, recruitment process and number of participants), type of vaccine (e.g., against SARS-CoV-2, measles, etc.), tool used to assess HL, outcome definition and measurement, statistical analysis, main findings, and adjustment factors. Two main outcomes were distinguished: intention to vaccinate and vaccination status. Articles were then grouped according to the type of vaccine and a narrative synthesis was performed for each outcome. Three independent authors performed a quality assessment of the articles included using the Newcastle-Ottawa scale for cohort studies or its adapted version for cross-sectional studies [30] (Supplementary Table S2). Discrepancies were resolved by consensus. Articles were considered of high quality when the total score was ≥7, fair quality if the score was ≥5 and <7, and poor quality if the score was lower than 5.

## 3. Results

**Figure 1:**
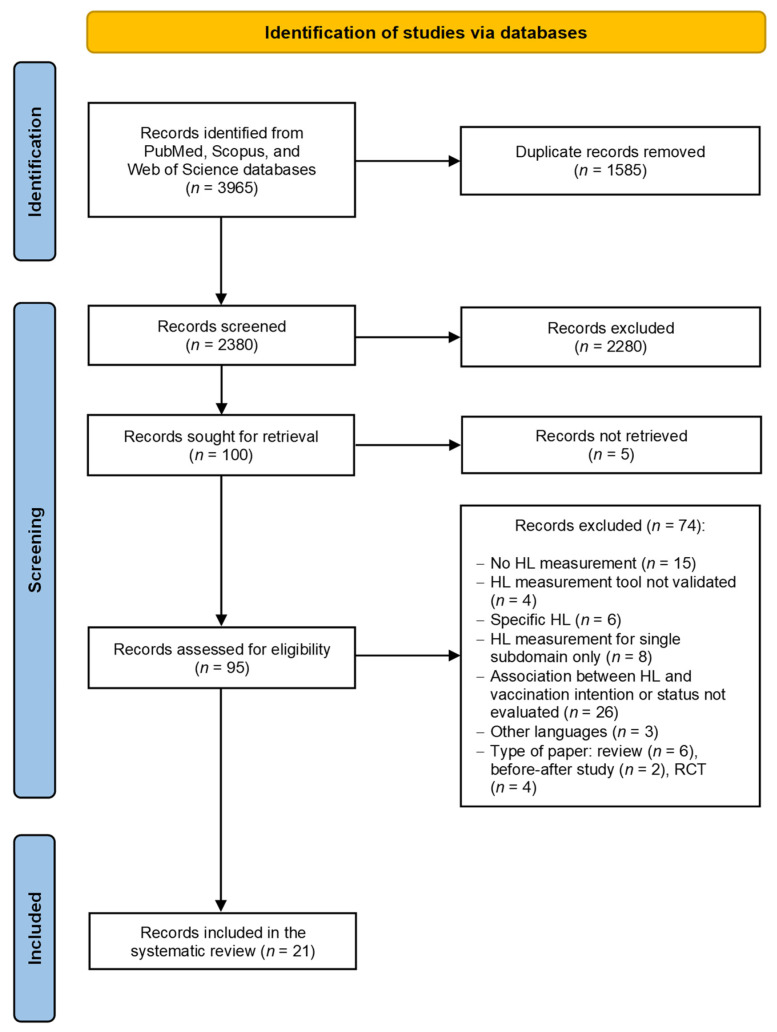
PRISMA flow diagram of the review process. HL: health literacy. RCT: randomized controlled trial.

Overall, 3965 records were identified by database searching (Figure 1). After duplicate removal and screening by title and abstract, 95 articles were selected as eligible for full-text analysis, from which 74 were excluded with reasons, giving a total of 21 articles ultimately included in the systematic review. Of these, six articles investigated intention to vaccinate [31,32,33,34,35,36], 14 records explored vaccination status [37,38,39,40,41,42,43,44,45,46,47,48,49,50], and one study [51] combined the two outcomes in a single analysis. In this last example [51], the composite outcome resulted from three questions, two of which referred to the vaccinations received in the previous years. For the purposes of this review, therefore, we considered this study to be an investigation of vaccination status.

### 3.1. Characteristics of the Studies Included

#### 3.1.1. Intention to Vaccinate

All studies investigating intention to vaccinate were published in 2021 and had a cross-sectional design (Table 1). Two were conducted in the United States [32,36], two in France [34,35], one in Japan [33] and one in Turkey [31]. In all studies but one [35], the authors specified the main characteristics of the target population: in one study, patients with chronic diseases were recruited from health clinics [32], while in three studies individuals were enrolled from educational settings (i.e., students or educators) [31,33,36], and in one study individuals attending homeless shelters were investigated [34]. About half the studies enrolled a large number of participants (i.e., more than 1000) [31,33,35]. All studies explored the intention to vaccinate against SARS-CoV-2. Quality was high in all cases except for one article [35], in which a lack of justification for the sample size and comparability between responders and non-responders were the main deficits (Supplementary Table S2).

#### 3.1.2. Vaccination Status

The articles that investigated vaccination status were published from 2002 to 2020 (Table 2). The majority were conducted in the United States (*n* = 10) [37,38,39,41,42,43,44,45,47,48], two in Europe (Italy and Spain) [49,51], two in Asia (South Korea and Malaysia) [40,50] and one in Israel [46]. They mostly had a cross-sectional design (*n* = 10) [37,40,41,43,44,47,48,49,50,51], while three were cohort studies (prospective or retrospective) [38,39,42,45] and one was a case–control study [46]. The target populations were heterogeneous, comprising parents or caregivers (*n* = 4) [42,45,46,48], pregnant women (*n* = 1) [49], healthcare workers (*n* = 2) [40,51], the elderly (*n* = 3) [37,38,44], and patients with chronic conditions (*n* = 2) [39,41]. The remaining three studies recruited adults [43,47,50], in one case only women [47]. Data were obtained from people seeking medical care in six studies [39,42,45,47,48,49]. Ethnicity was specified in two cases [47,50]. Six studies enrolled more than 1000 individuals [37,38,41,43,44,47], one of which included more than 10,000 participants [43]. Seven articles considered a single vaccination [40,41,44,47,48,50,51], five investigated two vaccines or more [37,38,39,43,49], and in three cases the authors explored combined vaccinations [42,45,46]. As a result, influenza vaccination was the most investigated (*n* = 11) [37,38,39,40,41,43,44,47,49,50,51] followed by pneumococcal (*n* = 4) [37,38,39,43]. Other vaccines included were hexavalent [45], a combination of hexavalent, measles, mumps and rubella (MMR), and pneumococcal [42], and diphtheria, tetanus and pertussis (DTPa) together with hepatitis B (HBV) and MMR [46], HPV [48] and pertussis [49]. Quality was generally quite high apart from two cases [50,51] that lacked a sample size justification and evidence of comparability between responders and non-responders (Supplementary Table S2).

### 3.2. Association between HL and Vaccination Behavior

#### 3.2.1. Intention to Vaccinate

HL was assessed using self-reported comprehension items in all but one study [31,33,34,35,36], which used a tool with reading comprehension and numeracy items (i.e., Newest Vital Sign) [32]. The HL level was then used in the analysis as a mean score in two cases [31,33], was categorized into two classes in two studies [35,36] or into three classes in the remaining study [32] (Table 3). The intention to be vaccinated was generally explored with one question on attitude and willingness to receive the COVID-19 vaccine [33,34,35,36], and was expressed as a scale in half the studies [31,32,33] or as a categorization in the remaining three articles [34,35,36]. Accordingly, three studies performed multivariable linear regressions or ANOVA [31,32,33], while the other three used logistic regression or its extension [34,35,36]. Results were inconsistent: HL seemed not to influence the intention to be vaccinated in three cases [31,32,36], whereas a significant association was found in two articles [33,34], with low HL levels predicting vaccine hesitancy in one case [34] and higher HL associated with vaccination intention in the other [33]. Lastly, one study [35] recorded a significant association between poor HL and vaccination intention, but only when comparing vaccine-hesitant and pro-vaccination individuals. All studies but one [32] conducted multivariable analyses; adjustment factors included were mainly socio-demographic characteristics.

#### 3.2.2. Vaccination Status

Among studies investigating influenza vaccination, the instruments used to measure HL were heterogeneous, but mostly used reading or numeracy comprehension items [37,38,39,42,43,44,48,49,50,51] (Table 4). In just one case, the authors assessed HL using three different tools [49]. HL was categorized into two or three levels in approximately half the studies [37,38,39,41,47,50]. Influenza vaccine uptake was explored using one or more self-reported questions in all studies but one, in which the immunization status was extracted from a registry [49]. The vaccination uptake was evaluated variously in the previous year [39,41,43,44,50], in one or more specific periods [40,49,51], or across the whole life of the individual [37,38], whereas Lorini et al. used a combination of questions on vaccination status and intention to vaccinate [51]. Vaccination uptake was expressed as a binary variable in almost all studies [37,38,39,40,41,43,44,47,49,50]. Results were contrasting: after adjusting mostly for socio-demographic, health status and health habit factors, inadequate but not marginal HL was strongly associated with vaccination refusal in the samples analyzed by Scott et al. [37] and Howard et al. [38]; low HL levels seemed to positively influence vaccination uptake in people aged less than 40 years and negatively influence it among people older than 65 years in one case [43]; high HL levels were significantly associated with vaccine uptake in two studies [44,50], whereas no relationship between HL and immunization status was obtained in five analyses [39,40,41,47,51]. Lastly, the study that used different tools to investigate the outcome found a significant association between high HL levels and vaccination uptake in one case out of three [49], but the analyses were unadjusted.

As for pneumococcal vaccine, all but one study [43] used the short version of the Test of Functional Health Literacy in Adults (S-TOFHLA) as the HL assessment tool, which groups HL into two [39] or three categories [37,38]. The other article used the National Assessment of Adult Literacy questionnaire. All studies investigated vaccination status with a self-reported question, at least once in the entire life [37,38,39] or during the previous year [43]. The outcome was always dichotomized into yes or no, and all articles provided adjusted estimates, using either logistic [37,38,39] or probit regression models [43]. No significant relationship was reported between HL and vaccination status except in one case [37] in which inadequate HL was associated with no vaccination uptake. Adjustment factors comprised mainly socio-demographic variables and health conditions.

HPV vaccination status among girls was assessed in one study [48] in which the authors used the Rapid Estimate of Adult Literacy in Medicine to categorize parents’ or caregivers’ HL levels into three classes. The outcome was calculated as time to completion of three out of four vaccine doses and was divided into four categories in relation to the delay in completion: not delayed (≤12 months), delayed 12–24 months, delayed 24–36 months and delayed >36 months. A multinomial logistic regression analysis found no association between any HL level and the delay in completion of HPV doses for any of the interval times considered. The analysis was adjusted mainly for socio-demographic characteristics of caregivers and the target population.

The current vaccination status of children for hexavalent vaccination was investigated by Pati et al. [45], who used S-TOFHLA to classify the HL of mothers as ‘adequate’ or ‘inadequate or marginal’, whereas data on vaccination status were extracted from an immunization registry. After adjustment mainly for the mothers’ socio-demographic variables, HL did not seem to predict the decision of mothers to vaccinate their children at three or seven months, according to multivariable logistic regression models.

Likewise, when exploring a similar population using the same HL tool several years later, but investigating the combination of hexavalent vaccine, MMR and pneumococcal vaccine, Pati et al. [42] did not find any relationship between maternal HL levels and the up-to-date immunization status of their children at 24 months, according to a univariable analysis.

The combined DTPa, MMR and HBV vaccination status was considered by Amit Aharon [46] using the Vaccine Health Literacy Scale to assess parents’ HL, calculating its level as a continuous variable. With immunization data extracted from a registry, the authors performed a path analysis and found a direct effect between parents’ communicative HL and the completion of the childhood vaccination protocol by the age of two, as well as an indirect effect between functional and critical HL and the same vaccination protocol.

Lastly, none of the three HL tools used by Castro-Sanchez et al. [49] detected any difference in the mean HL value of new mothers and pertussis vaccination received during pregnancy, according to a univariable analysis.

## 4. Discussion

The COVID-19 pandemic has rekindled interest in the importance of the population’s adherence to immunization programs and, consequently, the need to identify factors associated with vaccination uptake [52]. Among such factors, we investigated HL, which is broadly considered a social determinant of health [21,53,54] and a driver of healthy behavior [22,55], but we did not find conclusive evidence of its influence on the vaccination decision-making process. Because vaccination intention does not always reflect real behavior, and predictors might differ between the two aspects [56,57], we distinguished intention to vaccinate from vaccination status. However, in line with the mixed evidence provided by a previous review [23], our findings were largely inconsistent for both outcomes, probably due to the high degree of heterogeneity in the methods used. The issue of the multitude of tools commonly used to quantify HL [58], and accordingly the different domains explored [59], is widely discussed in the literature. In our review, we also found that different tools were used by researchers, and only some measured the individual’s capacity to read and understand actual material, minimizing the risk of an inaccurate self-assessment [17]. In this context, the development of a comprehensive instrument for HL evaluation is surely a challenge, but it would definitely enable a more precise estimation of the magnitude of the problem and a better comparison of evidence, even though HL remains strongly connected with cultural and social aspects that make it difficult to isolate this concept [17]. There are similar concerns about the measurement of outcomes, as recently demonstrated in a review that found different rates of vaccination acceptance according to the scale used to quantify COVID-19 vaccination intention [1]. The high degree of variability in the definition of vaccination status, which includes being up-to-date with vaccinations in the last few years, undergoing vaccination at least once over the individual’s entire life, or delaying the completion of a vaccination cycle, was also a concern. In addition, the cross-sectional design adopted in most studies complicates the causal interpretation of the findings [23]. For these reasons, to help clarify the role of HL in the vaccination decision-making process, future research on the topic should devise a longitudinal approach with a standardized methodology for the definition and measurement of both exposure and outcomes [23].

Apart from these general methodological considerations, the studies we found on intention to vaccinate focused exclusively on COVID-19 vaccines, probably because researchers wanted to investigate perceptions and intentions regarding newly developed vaccines administered during an out-of-the-ordinary campaign [60]. Our results suggest that factors other than HL are likely to explain people’s beliefs and intentions towards COVID-19 vaccines [31,32], such as trust in the government and institutions [32]. For this reason, communication strategies aimed at increasing public confidence in health authorities and helping people understand why recommended measures are useful to them and their community may be the most effective in promoting COVID-19 vaccine acceptance [61]. However, given the emergency context in which these surveys were conducted, more studies are needed to clarify the role of HL in the intention to vaccinate against SARS-CoV-2, but also against other diseases, particularly as, in the latter case, we were unable to find any relevant studies. In addition, to avoid polarizing the discussion around vaccines [60], future studies should differentiate between those who are hesitant and those who are openly against vaccination, as the determinants of the intention to vaccinate may be different in these two subgroups [62,63,64].

Besides the individual determinants that play a role in vaccination intention [65], a few factors may be critical in the actual administration of the injection [66], such as the availability and proximity of vaccination centers [66], the ease with which an appointment can be made [12], or the funding/reimbursement scheme [67]. In our review, the articles that investigated vaccination status mostly explored the determinants of influenza vaccination, probably because it is broadly recommended for the general population, there is an annual immunization program that struggles to reaches the desired coverage threshold, and there are huge variations in the uptake rates according to age and ethnicity [68]. Notably, the fact that a weak but positive association between HL and influenza vaccination uptake was mostly found in individuals aged more than 65 years [37,38,43,44,50] may be explained by the increased vulnerability of this age group to severe influenza outcomes, a factor often mentioned in the routine promotion campaigns that may encourage literate individuals to adhere to the recommended vaccination program [69]. In addition, the annual publicity for influenza vaccination delivery programs may promote vaccination uptake, which is different to what happens for pneumococcal vaccination, where there are less-widespread campaigns and the population’s perceived risk is particularly low [67]. As for the few studies focusing on pediatric and adolescent vaccinations, since we did not find consistent association between vaccination status and parents’ HL [42,45,46,48], more studies should be undertaken to investigate what influences vaccination uptake, especially considering that vaccine hesitancy in parents has contributed to the recent increase in vaccine-preventable disease outbreaks registered worldwide over recent years [9,70,71]. Furthermore, given that poor communication with parents was likely responsible for the association between vaccination rejection and a high level of education [49], increased attention should be given to communication strategies targeted to this particular group [72]. Specifically, tailored instruments and informative content that takes into account opinions, feelings and gaps in knowledge of the different vaccinations should be devised [73].

This study has some strengths and limitations. Firstly, we included observational studies that provided a general assessment of HL or that investigated all aspects of HL, excluding articles that analyzed only specific sub-domains. Nevertheless, given that we included articles that measured HL through validated and widely implemented tools, the resulting HL estimates can be considered reliable in relation to the multifaceted nature of the concept. Secondly, since our focus was HL generally, we excluded articles that investigated specific HL (e.g., cancer literacy, oral literacy). The other limitations are mostly related to the primary studies included in this review. Heterogeneity in the coding and measurement of HL and outcomes was found, largely limiting the opportunity to provide a quantitative synthesis. In addition, since our results are mostly based on self-reported outcomes, social desirability bias could affect the accuracy of our conclusions. Furthermore, since most studies were from the United States, and several of them analyzed specific subgroups, further research should be conducted both at the regional and national level to improve the generalizability of the findings. Nevertheless, to the best of our knowledge, this is the first review to perform an up-to-date systematic collection of evidence on the topic, expanding the findings provided in a previous review [23]. As a result, we were able to include information on COVID-19 vaccination. In addition, we were able to synthesize evidence on two different aspects of the vaccination decision-making process, namely, intention to vaccinate and vaccination status.

## 5. Conclusions

This review summarizes the current evidence on HL and intention to vaccinate and vaccination status. Despite some weak but positive results for influenza vaccination uptake in individuals aged more than 65 years, the relationship between HL and vaccination behavior remains scarcely supported by evidence. Differences in the methods used may explain the inconsistencies we found. Further research using a standardized approach is needed to clarify the role of HL in the vaccination decision-making process.

## Figures and Tables

**Table 1:** Characteristics of the studies on intention to vaccinate included in the systematic review.

First Author, Year	Country	Study Design	Target Population	Vaccination	Study Quality
Aslantekin-Özçoban, 2021	Turkey	CS	Midwifery students (N = 1891)	SARS-CoV-2	8
Arvanitis, 2021	USA	CS	Older adults with one or more chronic diseases recruited from a community of academic clinics (N = 601)	SARS-CoV-2	8
Fukuda, 2021	Japan	CS	School, college and university educators aged 20–60 years (N = 1000)	SARS-CoV-2	9
Longchamps, 2021	France	CS	Homeless people aged ≥18 years recruited from short and long-term homeless shelters (N = 235)	SARS-CoV-2	7
Montagni, 2021	France	CS	Adults >18 years from the general population enrolled from PBS (N = 1647)	SARS-CoV-2	6
Patil, 2021	USA	CS	College students (N = 256)	SARS-CoV-2	8

CS: Cross-sectional. HL: Health Literacy. US: United States. PBS: population-based study.

**Table 2:** Characteristics of the studies on vaccination status included in the systematic review.

First Author, Year	Country	Study Design	Target Population	Vaccination	Study Quality
Scott, 2002	US	Cross-sectional	Community-dwelling Medicare aged ≥65 years enrolled in a national managed care organization (N = 2722)	Influenza, pneumococcal	8
Howard, 2006	US	Retrospective cohort	Community-dwelling Medicare aged ≥65 years enrolled in a national managed care organization (N = 3260)	Influenza, pneumococcal	8
White, 2008	US	Cross-sectional	Individuals aged ≥16 years enrolled from PBS (N = 18,100)	Influenza, pneumococcal	8
Bennett, 2009	US	Cross-sectional	Individuals aged ≥65 years enrolled from PBS (N = 2668)	Influenza	9
Pati, 2010	US	Prospective cohort	Medicaid-eligible mothers recruited in urban hospital (N = 506)	Hexavalent	9
Amit Aharon, 2017	Israel	Case-control	Parents recruited from a health registry (N = 731)	DTPa + HBV + MMR	7
Moran, 2017	US	Cross-sectional	Hispanic women aged between 21 and 50 years recruited in clinics and community sites (N = 1565)	Influenza	7
Pati, 2017	US	Prospective cohort	Medicaid-eligible mothers recruited in urban hospital (N = 693)	Hexavalent + MMR + pneumococcal	8
Widdice, 2018	US	Cross-sectional	Caregivers enrolled at time of the third dose vaccination recruited in pediatric adolescent and family medicine practices (N = 422)	HPV	8
Castro-Sánchez, 2018	Spain	Cross-sectional	Pregnant women recruited in maternity wards (N = 119)	Influenza, pertussis	9
Song, 2018	South Korea	Cross-sectional	Adult North Korean defectors (N = 399)	Influenza	6
O’Conor, 2019	US	Prospective cohort	Adults with COPD recruited in a community clinic (N = 388)	Influenza, pneumococcal	8
Rafferty, 2019	US	Cross-sectional	Adult diabetic patients (N = 4397)	Influenza	8
Omar, 2020	Malaysia	Cross-sectional	Healthcare workers (N = 775)	Influenza	10
Lorini, 2020 ^a^	Italy	Cross-sectional	Healthcare workers (N = 711)	Influenza	6

^a^ This study combined questions on vaccination status and intention in a single outcome. DPTa: diphtheria, tetanus, pertussis. HBV: hepatitis B. HPV: human papillomavirus. MMR: measles, mumps, rubella. PBS: population-based study. COPD: chronic obstructive pulmonary disease.

**Table 3:** Association between health literacy (HL) and vaccination intention against SARS-CoV-2.

Author, Year	HL		Vaccination Intention		Statistical Analysis	Main Findings	Adjustment Factors
	Measurement ^α^	Coding	Measurement	Coding			
Aslantekin-Özçoban, 2021	HLS-EU-Q25	Continuous	Nine questions investigating trust, perceived effectiveness, and benefits of the COVID-19 vaccination	Vaccination attitude scale (score: 0–5): - low score: negative attitude - high score: positive attitude	Multivariable linear regression	Non-significant association between HL and vaccination attitude (aβ = 0.027, *p* = 0.188)	Attitudes towards COVID-19 vaccine; perception of COVID-19 causes
Arvanitis, 2021	NVS	Three categories: - low - marginal - adequate	Two questions: - “*I trust that any future coronavirus vaccine will be safe and effective*” - “*I will be vaccinated for the coronavirus as soon as a vaccine becomes* *available*”	Vaccination agreement scale (score: 0–10): - low score: no agreement - high score: total agreement	ANOVA	Non-significant association between HL and vaccination agreement (*p* = 0.06)	/
Fukuda, 2021	HLS-EU-Q47	Continuous	One question relating to the timing of vaccination intention after inoculation availability	Vaccination intention scale (score: 1–5): - low score: will not inoculate - high score: immediately	Multivariable linear regression	Significant association between higher HL and vaccination intention (aβ = 0.021, *p* < 0.001)	Gender; age; academic background; being under care of physician
Longchamps, 2021	HLQ	Two categories: - low - intermediate or high	One question: *“If a vaccine* *existed, would you be willing to get vaccinated?”*	Two categories of vaccination intention: - yes: not hesitant - no or I don’t know: vaccine hesitant	Multivariable logistic regression	Significant association between intermediate/high HL and vaccine hesitancy (aOR = 0.38, 95% CI: 0.21–0.68)	Gender; family composition; administrative status
Montagni, 2021	5-item scale developed by the French Public Health Agency	Two categories: - bad - good	One question: *“Would you be willing to get vaccinated against* *coronavirus even if the vaccine* *has not yet been fully proven* *effective?”*	Three categories of vaccination intention: - yes: pro-vaccination - I don’t know: vaccine hesitant - no: anti-vaccination	Multinomial logistic regression	- Significant association between poor HL and being vaccine hesitant vs. pro-vaccination (aRRR = 1.44, 95% CI: 1.04–2.00) - Non-significant association between poor HL and being anti-vaccination vs. pro-vaccination (aRRR = 1.25, 95% CI: 0.96–1.63)	Gender; being regularly vaccinated against the flu; having an up-to-date vaccination; studying or working in the health domain, capacity to detect fake news
Patil, 2021	SILS	Two categories: - low - adequate	One question: *“How likely would you be to get a COVID-19 vaccine, if* *available?”*	Two categories of vaccination intention: - very likely: willing to undergo vaccination - somewhat or not likely: vaccine hesitant	Multivariable logistic regression	Non-significant association between low HL and likelihood to vaccinate (aOR = 0.88, 95% CI: 0.50–1.56)	Social network size; gender; race/ethnicity; disability; first generation university student status; political affiliation

aβ: adjusted beta coefficient. aOR: adjusted odds ratio. aRRR: adjusted relative risk ratio. ANOVA: analysis of variance. CI: confidence interval. COVID-19: coronavirus disease 2019. ^α^ HL tool: HLQ: Health literacy Questionnaire. HLS-EU-Q: European Health Literacy Survey Questionnaire. NVS: Newest Vital Sign. SILS: Single Item Literacy Screener.

**Table 4:** Association between health literacy (HL) and vaccination status by vaccine type.

Author, Year	HL		Vaccination Status		Statistical Analysis	Main Findings	Adjustment Factors ^β^
	Measurement ^α^	Coding	Measurement	Coding			
Influenza							
Scott, 2002	S-TOFHLA	Three categories: - inadequate - marginal - adequate	One SR question: having ever received the vaccination	Two categories of vaccination status: - yes - no	Multivariable logistic regression	- Significant association between inadequate HL and no vaccination uptake (aOR = 1.4, 95% CI: 1.1–1.9) - Non-significant association between marginal HL and no vaccination uptake (aOR = 1.0, 95% CI: 0.7–1.4)	AGR, education, income, physician visit (last 3 months), MMSE, chronic condition, IADL limitation
Howard, 2006	S-TOHFLA	Three categories: - inadequate - marginal - adequate	One SR question: having ever received the vaccination	Two categories of vaccination status: - yes - no	Multivariable logistic regression	- Significant association between inadequate HL and vaccination uptake (aOR = 0.76, *p* = 0.020) - Non-significant association between marginal HL and vaccination uptake (aOR = 1.06, *p* = 0.707)	AGR, education, income, tobacco consumption, chronic conditions, area of residence
White, 2008	NAAL	Continuous	One SR question: having received the vaccination in the previous year	Two categories of vaccination status: - yes - no	Marginal maximum likelihood probit regression	- Adults aged <40 years: significant association between higher HL and vaccination uptake (aβ = −0.07, *p* < 0.05) - Adults aged 40–64 years: non-significant association between higher HL and vaccination uptake (aβ = 0.01, *p* > 0.05) - Adults aged >65 years: significant association between higher HL and vaccination uptake (aβ = 0.17, *p* < 0.05)	AGR, health status, poverty level, insurance coverage, oral reading fluency
Bennett, 2009	NAAL	Continuous	One SR question: having ever received the vaccination	Two categories of vaccination status: - yes - no	Marginal maximum likelihood probit regression	Significant association between higher HL and vaccination uptake (aβ = 0.14, *p* < 0.05)	AGR, education, income, US born
Moran, 2017	SBSQ	Two categories: - inadequate - adequate	One SR question: frequency of vaccination against influenza	Two categories of frequency of vaccination: - almost always or always: regularly receiving vaccination - never, rarely, or sometimes: not regularly receiving vaccination	Multivariable logistic regression	Non-significant association between adequate HL and regularly receiving influenza vaccination (aOR = 1.12, 95% CI: 0.88–1.43)	Country of birth, educational level, annual income, age, health insurance, health state, fatalism, acculturation, years lived in the US, religiosity, confidence in the vaccine safety
Castro-Sánchez, 2018	SAHLSA-50	Continuous	Immunization status extracted from the vaccination registry	Two categories of vaccination status: - received the vaccination during pregnancy - did not receive the vaccination during pregnancy	Mann–Whitney U test	Significant association between higher HL and vaccination uptake (*p* = 0.019)	/
	NVS	Continuous			Mann–Whitney U test	Non-significant association between higher HL and vaccination uptake (*p* = 0.320)	
	SILS	Continuous			Mann–Whitney U test	Non-significant association between higher HL and vaccination uptake (*p* = 0.942)	
Song, 2018	S-KHLS	Three categories: - low - intermediate - high	One SR question: having received the vaccination in the previous two years	Two categories of vaccination status: - yes - no	Multivariable logistic regression	- Significant association between intermediate HL and vaccination uptake (aOR = 2.44, 95% CI: 1.19–5.00) - Significant association between high HL and vaccination uptake (aOR = 2.10, 95% CI: 1.02–4.35)	Age, gender, marital status, duration of stay in other countries before entry, duration of stay in the Republic of Korea
O’Conor, 2019	S-TOFHLA	Two categories: - limited - adequate	One SR question: having received the vaccination in the previous year	Two categories of vaccination status: - yes - no	GEE model for repeated measurements	Non-significant association between adequate HL and vaccination uptake (aOR =0.85, 95% CI: 0.62–1.18)	AGR, income, number of comorbidities, severity of COPD
Rafferty, 2019	BRFSS	Two categories: - not low- low	One SR question: having received the vaccination in the previous year	Two categories of vaccination status: - yes - no	Multivariable logistic regression	Non-significant association between low HL and vaccination uptake (aOR = 0.98, 95% CI: 0.74–1.29)	AGR, education, household income, health status
Omar, 2020	FCCHL	Continuous	One SR question: *“Did you have an influenza vaccination* *between* *November 2016 and October 2017?”*	Two categories of vaccination status: - yes - no	Multivariable logistic regression	Non-significant association between higher HL and vaccination uptake: - functional HL: aOR = 1.04, 95% CI: 0.79–1.37 - critical HL: aOR = 1.08, 95% CI: 0.76–1.53 - communicative HL: aOR = 0.98, 95% CI: 0.65–1.45	AGR, religion, education, job category, department, income, chronic disease, marital status, smoking status, and living with person at high risk of getting influenza complications, knowledge, behavioral, HL variables
Lorini, 2020	IMETER	Continuous	Three SR questions: having received the vaccination in 2016–2017, in 2017–2018, and intention to vaccinate in 2018–2019	Three categories of vaccination behavior: - always get vaccinated - vaccinated at least once in the previous years or intended to vaccinate (sometimes) - never vaccinated and did not intend to vaccinate (never)	Multinomial logistic regression	- Non-significant association between higher HL and vaccination behavior (sometimes vs. never: aRRR = 0.99, 95% CI: 0.97-1-01) - Non-significant association between higher HL and vaccination behavior (always vs. never: aRRR = 0.98, 95% CI: 0.95–1.01)	Gender, age, mother language
Pneumococcal							
Scott, 2002	S-TOFHLA	Three categories: - inadequate - marginal - adequate	One SR question: having ever received the vaccination	Two categories of vaccination status: - yes - no	Multivariable logistic regression	- Significant association between inadequate HL and no vaccination uptake (aOR = 1.3, 95% CI: 1.1–1.7) - Non-significant association between marginal HL and no vaccination uptake (aOR = 1.2, 95% CI: 0.9–1.7)	AGR, education, income, physician visit (last 3 months), MMSE, chronic condition, IADL limitation
Howard, 2006	S-TOFHLA	Three categories: - inadequate - marginal - adequate	One SR question: having ever received the vaccination	Two categories of vaccination status: - yes - no	Multivariable logistic regression	- Non-significant association between inadequate HL and vaccination uptake (aOR = 0.85, *p* = 0.114) - Non-significant association between marginal HL and vaccination uptake (aOR = 0.91, *p* = 0.445)	AGR, education, income, tobacco consumption, chronic conditions, area of residence
White, 2008	NAAL	Continuous	One SR question: having received the vaccination in the previous year	Two categories of vaccination status: - yes - no	Marginal maximum likelihood probit regression	Non-significant association between higher HL and vaccination uptake (aβ = −0.01, *p* > 0.05)	AGR, health status, poverty level, insurance coverage, oral reading fluency
O’Conor, 2019	S-TOFHLA	Two categories: - limited - adequate	One SR question: having ever received the vaccination	Two categories of vaccination status: - yes - no	GEE models for repeated measurements	Non-significant association between adequate HL and vaccination uptake (aOR = 1.01, 95% CI: 0.64–1.60)	AGR, income, number of comorbidities, severity of COPD
HPV							
Widdice, 2018	REALM	Three categories of HL grade equivalent: - 6th grade or below (very low) - 7th to 8th grade (low) - high school	One SR question: time to completion of three doses (days between the first and third dose)	Four categories of time to completion of vaccination cycle: - not delayed (≤12 months) - delayed (12–24 months) - delayed (24–36 months) - delayed (>36 months)	Multinomial logistic regression	- Non-significant association between very low HL and delayed completion of vaccination cycle - Non-significant association between low HL and delayed completion of vaccination cycle	Race, caregiver education, adolescent insurance, gap in adolescent insurance since HPV dose, number of parents in household, parents’ marital status, adolescent health visits in the previous year, appointment availability, caregiver ability to obtain timely appointments for adolescent’s medical care, ability to get through on the telephone
Hexavalent							
Pati, 2010	S-TOFHLA	Two categories: - inadequate or marginal - adequate	Immunization status extracted from vaccination registry	Two categories for up-to-date vaccination status at 3 and 7 months: - yes - no	Multivariable logistic regression	Non-significant association between higher maternal HL and child’s up-to-date immunization status: - 3 months (aOR = 1.08, 95% CI: 0.67–1.76) - 7 months (aOR = 0.92, 95% CI: 0.57–1.48)	Maternal race/ethnicity, age, education, receiving antenatal care, participation in WIC program, marital status, location of the infant’s health care facility, vaccination status at the age of 3 months (for 7 months model only)
Hexavalent + MMR + Pneumococcal							
Pati, 2017	S-TOFHLA	Two categories: - inadequate or marginal - adequate	Immunization status extracted from vaccination registry	Two categories for up-to-date vaccination status at 24 months: - yes - no	Chi-square test	Non-significant association between maternal HL and child’s up-to-date immunization status at 24 months	/
DTPa + MMR + HBV							
Amit Aharon, 2017	VHLS	Continuous	Immunization status extracted from vaccination registry	Completion of vaccination protocol at 2 years: - yes - no	Path analysis	- Significant association between higher communicative HL and completion of vaccination protocol at 2 years (β = 0.06, *p* < 0.05) - Functional HL and critical HL had an indirect effect, mediated through other variables, on completion of the vaccination protocol at 2 years	NA
Pertussis							
Castro-Sánchez, 2018	SAHLSA-50	Continuous	Immunization status extracted from vaccination registry	Two categories of vaccination status: - received the vaccination during pregnancy - did not receive the vaccination during pregnancy	Mann–Whitney U test	Significant association between higher HL and vaccination uptake (*p* < 0.05)	/
	NVS	Continuous			Mann–Whitney U test	Non-significant association between higher HL and vaccination uptake (*p* > 0.05)	
	SILS	Continuous			Mann–Whitney U test	Non-significant association between higher HL and vaccination uptake (*p* > 0.05)	

aβ: adjusted beta coefficient. aOR: adjusted odds ratio. aRRR: adjusted relative risk ratio. CI: confidence interval. GEE: generalized estimating equation. DPTa: diphtheria, tetanus, pertussis. HBV: hepatitis B. HPV: human papillomavirus. MMR: measles, mumps, rubella. SR: self-reported. ^α^ HL tool: TOFHLA: Test of Functional Health Literacy in Adults. NAAL: National Assessment of Adult Literacy. VHLS: Vaccine Health Literacy Scale. SBSQ: Set of Brief Screening Questions. REALM: Rapid Estimate of Adult Literacy in Medicine. SAHLSA: Short Assessment of Health Literacy for Spanish Adults. NVS: Newest Vital Sign. FCCHL: Functional Communicative Critical Health Literacy. IMETER: Italian Medical Term Recognition Test. BRFSS: Behavioral Risk Factor Surveillance System. S-KHLS: Korean Health Literacy Scale. ^β^ Adjustment factors: AGR: Age, gender, race. COPD: Chronic obstructive pulmonary disease. MMSE: Mini Mental State Examination. IADL: Instrumental activities of daily living. WIC: Women, Infants, and Children.

## Data Availability

Not applicable.

## Supplementary Materials

The following supporting information can be downloaded at: https://www.mdpi.com/article/10.3390/vaccines10111832/s1, Table S1: Search strategies used in the systematic review; Table S2: Quality assessment of the articles included in the systematic review according to the Newcastle-Ottawa scale for cohort and case–control studies and its adapted version for cross-sectional studies.
