# Supplementary material for: The Association of Health Literacy with Intention to Vaccinate and Vaccination Status: A Systematic Review
Source: Vaccines (Basel). 2022 Oct 29;10(11):1832. doi: 10.3390/vaccines10111832 (PMC9692286; doi:10.3390/vaccines10111832)
Supplement: Supplementary file 1 [file vaccines-10-01832-s001.zip › vaccines-1979390-supplementary.pdf]

SUPPLEMENTARY MATERIAL

Supplementary Table S1. Search strategies used in the systematic review.

("health" AND "literacy") OR ("health literacy" OR "health literacy[MeSH Terms]") AND ("vaccin*" OR "immuniz*" OR "immunis*" OR "vaccination[MeSH Terms]" OR "vaccines[MeSH Terms]")	PubMed
("health" AND "literacy") OR ("health literacy") AND ("vaccin*" OR "immunis*" OR "immuniz*")	Scopus
("health" AND "literacy") OR ("health literacy") AND ("vaccin*" OR "immuniz*" OR "immunis*")	Web of Science

Supplementary Table S2. Quality assessment of the articles included in the systematic review according to the Newcas-tle-Ottawa scale for cohort and case–control studies and its adapted version for cross-sectional studies.

First author, year	Selection				Comparability	Outcome			Overall
Patil, 2021	1	1	0	2	2	1	1	-	8
Montagni, 2021	1	0	0	1	2	1	1	-	6
Longchamps, 2021	1	0	0	2	2	1	1	-	7
Fukuda, 2021	1	1	1	2	2	1	1	-	9
Arvanitis, 2021	1	0	1	2	2	1	1	-	8
Aslantekin-Özçoban, 2021	1	1	0	2	2	1	1	-	8
Scott, 2002	1	1	0	2	2	1	1	-	8
Howard, 2006	1	1	1	1	2	0	1	1	8
White, 2008	1	1	1	2	1	1	1	-	8
Bennett, 2009	1	1	1	2	2	1	1	-	9
Pati, 2010	1	1	1	1	2	1	1	1	9
Pati, 2017	0	1	1	1	2	1	1	1	8
Amit Aharon, 2016	1	0	0	1	2	2	1	-	7
Moran, 2016	0	0	1	2	2	1	1	-	7
Widdice, 2018	0	0	1	2	2	2	1	-	8
Castro-Sánchez, 2018	1	1	1	2	1	2	1	-	9
O'Conor, 2019	1	1	1	0	2	1	1	1	8
Omar, 2020	1	1	1	2	2	2	1	-	10
Lorini, 2020	1	0	0	1	2	1	1	-	6
Rafferty, 2019	1	1	0	2	2	1	1	-	8
Song, 2018	1	0	0	1	2	1	1	-	6
